# Diet associations in endometriosis: a critical narrative assessment with special reference to gluten

**DOI:** 10.3389/fnut.2023.1166929

**Published:** 2023-09-04

**Authors:** Fred Brouns, Annelotte Van Haaps, Daniel Keszthelyi, Koen Venema, Marlies Bongers, Jacques Maas, Velja Mijatovic

**Affiliations:** ^1^Department of Human Biology, School for Nutrition and Translational Research in Metabolism, Maastricht University, Maastricht, Netherlands; ^2^Endometriosis Center, Amsterdam University Medical Centers, Academic Medical Center, Amsterdam, Netherlands; ^3^Amsterdam Reproduction and Development Research Institute, Amsterdam, Netherlands; ^4^Division of Gastroenterology-Hepatology, Department of Internal Medicine, School for Nutrition and Translational Research in Metabolism, Maastricht University, Maastricht, Netherlands; ^5^Centre for Healthy Eating & Food Innovation (HEFI), Maastricht University, Maastricht, Netherlands; ^6^Department of Obstetrics and Gynecology, Máxima Medical Center, Veldhoven, Netherlands; ^7^Grow-School of Oncology and Reproduction, Maastricht University, Maastricht, Netherlands; ^8^Department of Obstetrics and Gynaecology MUMC+, Maastricht, Netherlands

**Keywords:** endometriosis, diet associations, diet recommendations, omega-3, red meat, gluten-free

## Abstract

Endometriosis is characterized by the presence of endometrium-like tissue outside the uterus. The etiology remains largely unknown. Despite adequate treatment, patients can still experience symptoms or side effects resulting in therapy incompliance and in self-management strategies such as dietary measures is increasing. A gluten free diet is thought to be contributory in reducing endometriosis-related pain, thereby optimizing quality of life. However, data is conflicting and currently provides no evidence for causality. This narrative review aims to put the effect of dietary self-management strategies on endometriosis in a balanced perspective, especially the effect of gluten and a gluten free diet. Several studies have found a strong overlap in symptoms, metabolic and immune responses associated with endometriosis and those associated with celiac disease, ulcerative colitis, Crohn’s disease, irritable bowel syndrome and non-celiac wheat sensitivity. However, it remains unclear whether these diseases and/or disorders are causal to an increased risk of endometriosis. Some studies have found a positive effect on the risk of endometriosis, endometriosis-related symptoms and quality of life (QoL) when women either avoided certain nutrients or foods, or applied a specific nutrient supplementation. This includes the avoidance of red meat, an increasing intake of foods rich in anti-oxidants, omega-3, micronutrients and dietary fibers (e.g., fruit, vegetables) and the appliance of a gluten free diet. However, data from the available studies were generally graded of low quality and it was noted that placebo and/or nocebo effects influenced the reported positive effects. In addition, such effects were no longer seen when adjusting for confounders such as overweight, when a translation was made from *in vitro* to *in vivo*, or when the nutrients were not supplemented as isolated sources but as part of a mixed daily diet. Finally, some studies showed that long-term adherence to a gluten free diet is often associated with an impaired diet quality and nutrient intake, leading to negative health outcomes and reduced QoL. Concluding, scientific evidence on the efficacy of dietary interventions on well-defined clinical endpoints of endometriosis is lacking and recommending a gluten free diet to women solely diagnosed with endometriosis should therefore not be advised.

## Introduction

Endometriosis is characterized by the presence of endometrium-like tissue outside the endometrium and myometrium, usually with an associated inflammatory process (1). The presence of intra-abdominal endometriosis can be suspected based on clinical symptoms such as dysmenorrhea, dyschezia, hematochezia, dysuria, hematuria, dyspareunia, chronic pelvic pain and infertility. Complaints are often related to the menstrual cycle and may be progressive in nature. Therefore, endometriosis can be associated with reduced mental, physical and social wellbeing leading to a lower quality of life (QoL) (2–4). Because of this, Saunders and Horne (5) recently proposed to consider endometriosis as a syndrome rather than a single disease state. The QoL impairment, the risk of work-related disability and unemployed work status as well as the related costs are high and similar to other widespread chronic inflammatory conditions such as Diabetes Mellitus Type 2, Crohn’s disease and Rheumatoid Arthritis (1, 3, 6, 7).

**Figure 1:**
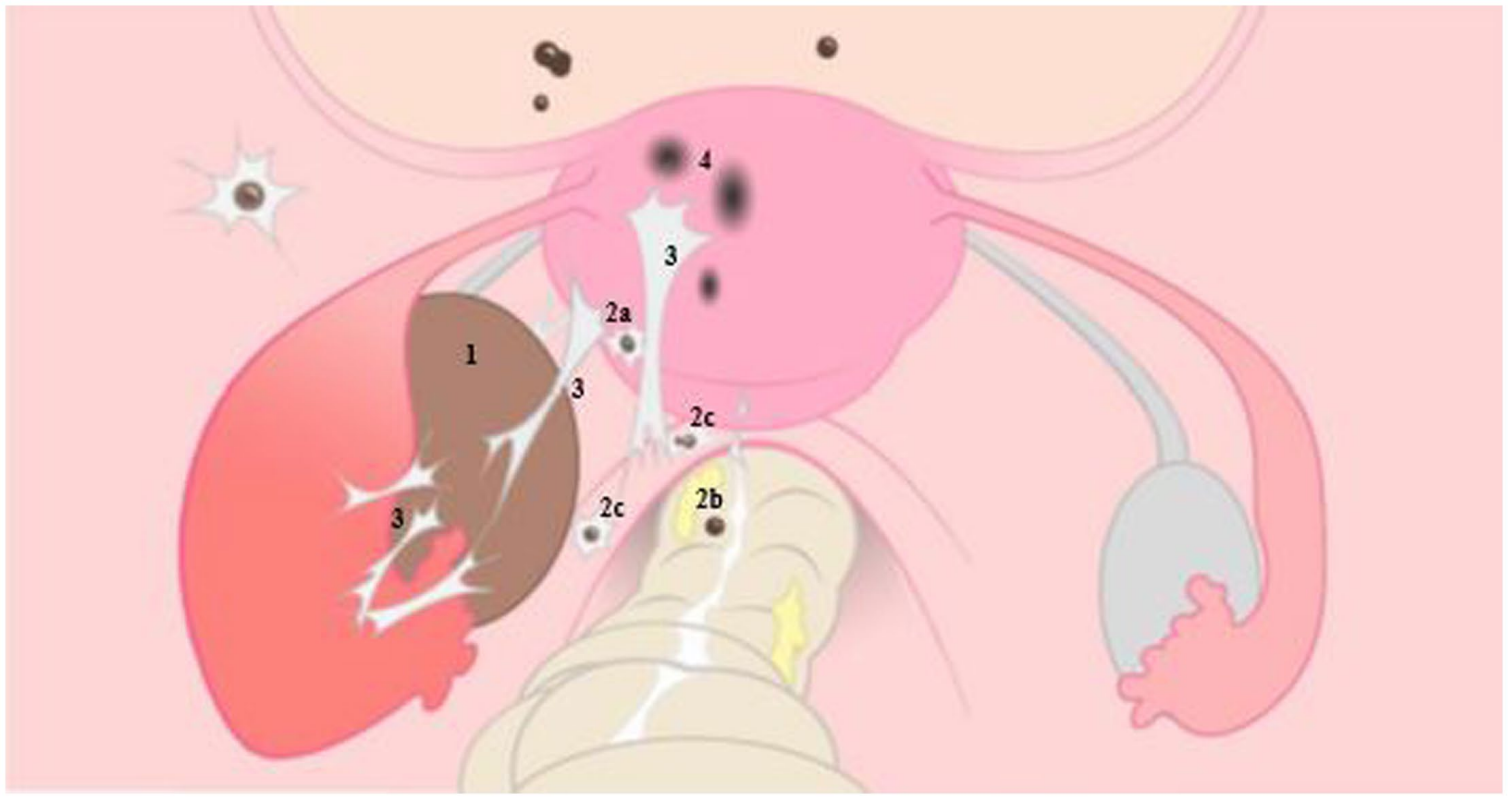
Illustrative representation of endometriosis on the left, with normal anatomy of the female reproductive organ on the right. 1: ovarian endometrioma, 2: deep endometriosis located on the bladder and posterior uterine wall (2a), on the rectal wall (2b) and sacro-uterine ligament (2c), 3: adhesions caused by endometriosis, 4: adenomyosis. With permission L. van der Houwen (8).

Because many endometriosis symptoms strongly overlap with other chronic conditions such as irritable bowel syndrome (IBS), inflammatory bowel disease (IBD) and celiac disease (CD) (see Figure 1), early diagnosis is often missed (9–16). In the Netherlands it may take up to 10 years, with a median delay of 7.4 years between a first visit to a medical practitioner and a confirmed diagnosis of endometriosis (17). During the period of diagnostic delay, 75% of women consult 1–4 medical professionals for their symptoms because they are not satisfied with the advice and/or treatment offered (18). In general, it can be stated that clinical symptoms and the experience of the patient do not always reflect the severity of the disease (19). Treatment of endometriosis consists of four pillars: hormonal therapy, pain management, surgery and/or therapy with assisted reproductive techniques (ART). Nevertheless, applied medical and/or surgical treatment methods can be insufficient to alleviate symptoms or may be accompanied with side effects. Both can affect treatment compliance negatively (20–22). This has resulted in an increasing interest in self-management strategies among women diagnosed with endometriosis (23).

Within the realms of complementary and alternative medicine suggestions are being made, i.e., on social media but also in a number of medical publications, that a range of non-medical, often self-management strategies might result in pain relief and an improvement of wellbeing. They include relaxation exercises (e.g., yoga, breathing exercises and meditation), the use of heat, acupuncture, physiotherapy and the use of cannabidiol (CBD) oil. In addition, specific dietary measures such as a gluten free diet (GFD), the Low FODMAP diet and the so-called endometriosis diet (aspects of these are discussed further below in detail) have been suggested to help mitigate endometriosis associated pain and discomfort (1, 24–30). Women that are receptive to suggestions regarding symptom reduction often self-implement such measures (23, 26, 31–35) and recently it was shown that this may result in positive effects on QoL (36, 37). Because of this, the current European endometriosis guidelines advice healthcare providers to discuss non-medical strategies and address aspects of QoL and psychosocial wellbeing (1). However, since evidence providing plausible biochemical mechanisms explaining the perceived benefits is scarce or lacking (30) no recommendations are made in these guidelines regarding dietary strategies (1).

Associations between diet components and endometriosis have been addressed in a number of recent systematic analysis and reviews but they do not reflect causality (38–44). Plausible mechanisms related to the effects of specific food components on pathology and symptoms *in vivo*, that may underlie these associations, have thus far hardly been discussed thoroughly. Much of the data pointing to plausible mechanisms is obtained from *in vitro* and animal studies which do not necessarily reflect the human situation. Therefore, we aim to critically discuss the explaining associations between endometriosis and dietary interventions, and their challenges and limitations. We will do this by the example of three selected diet components. Supplementation with omega 3 fatty acids and red meat avoidance will be discussed in short, whereas the association between gluten related pathologies and endometriosis will be discussed in great detail.

For this narrative overview, selected original research papers were obtained using the university E-Library academic search tools PUBMED, WEB OF SCIENCE, SCOPUS and EMBASE, ENDNOTE global search tool, as well reference lists in research papers and reviews (see Appendix). Papers on randomized controlled trials, retrospective and prospective studies, systematic reviews and meta-analysis were included in this narrative review. Relevant citations in publications were cross-checked with the original content in the sources cited.

### Endometriosis background

#### Prevalence of endometriosis

It is estimated that over 175 million women worldwide suffer from endometriosis and is thought to occur in 5%–15% in women of reproductive age (15–49 years) (45–50). With women of reproductive age representing about 38% of the global population, the currently reported endometriosis prevalence equals between 1.9% and 5.7% in the global population (51, 52). Underreporting due to diagnostic complexity and delay is very likely.

#### Causes and mechanisms associated with endometriosis

Due to the complexity of endometriosis and the multiple associations with other diseases and/or disorders, many hypotheses on endometriosis etiology have been put forward. The most accepted and oldest theory dating back to 1927 is the “Retrograde menstruation” theory, also referred to as the “Implantation Theory”. In this theory it is proposed that fragments of the endometrium are dragged through the Fallopian tubes into the abdominal cavity during menstruation, followed by implantation on the peritoneum. These mucosal fragments can form endometriotic lesions which in turn can spread through the abdominal cavity. However, it should be noted that the phenomenon of retrograde menstruation physiologically occurs in the majority of women with patent Fallopian tubes whereas only a fraction of women develop endometriosis (53–55). Therefore, other theories are proposed including coelomic metaplasia (which has been suggested in women with Mullerian duct defects and explains peritoneal lesions by the transformation of peritoneal mesothelium into glandular endometrium) and hematologic or lymphatic spread (which explains extra-pelvic lesions) (5, 22).

The pathogenesis of endometriosis includes local estrogen production, progestogen resistance as well as chronic local and systemic inflammation. An altered inflammatory response, usually mediated by tumor necrosis factor (TNF)-alpha expression and sustained by Interleukin (IL)-16 in the peritoneal cavity, appears to be estrogen-dependent. Post-menarchal increased estrogen levels have been shown to impact endometriosis prevalence, whereas reduced levels are clearly associated with a lower prevalence (56). In addition, estrogen contributes to the growth and proliferation of endometrial cells, both *in utero* and of ectopic endometriosis lesions. It also suppresses the expression of several anti-inflammatory genes (IL-8, IL-6, TNF-a) and is involved in the modulation of endometriosis-related pain by exerting neuro-modulatory functions, both through indirect and direct mechanisms, on sensory and sympathetic nerves (57). Suggestions have been put forward that estrogens from meat consumption may contribute to endometriosis risk resulting in recommendations to avoid or limit red meat consumption (58, 59). Finally, another theory concerns “Bacterial /microbial contamination and ‘Dysbiosis’^1^” (61–64). It is suggested that intestinal dysbiosis, for example in celiac disease patients, is associated with inflammation, impaired barrier function and microbial translocation possibly increasing the risk of endometriosis. However, thus far no plausible mechanisms have been defined to explain how dysbiosis could cause endometriosis. Below we discuss this matter in more detail. Extensive detail on endometriosis subtypes, pathogenically mechanisms and etiology pathways can be found in recent reviews (5, 55, 65–68). An illustrative representation of endometriosis is given in Figure 1.

### Critical assessment and discussion on the association and cause-effect of selected dietary factors in endometriosis

Several studies have suggested there might be a causal effect between the development of endometriosis and either supplementation or avoidance of certain supplements and/or nutrients. However, these studies are often of low quality and only show associations. They do not provide proof of causality. Therefore, a number of critical aspects in the assessment of the effect of a diet or specific dietary components on health outcomes are discussed below.

**Figure 2:**
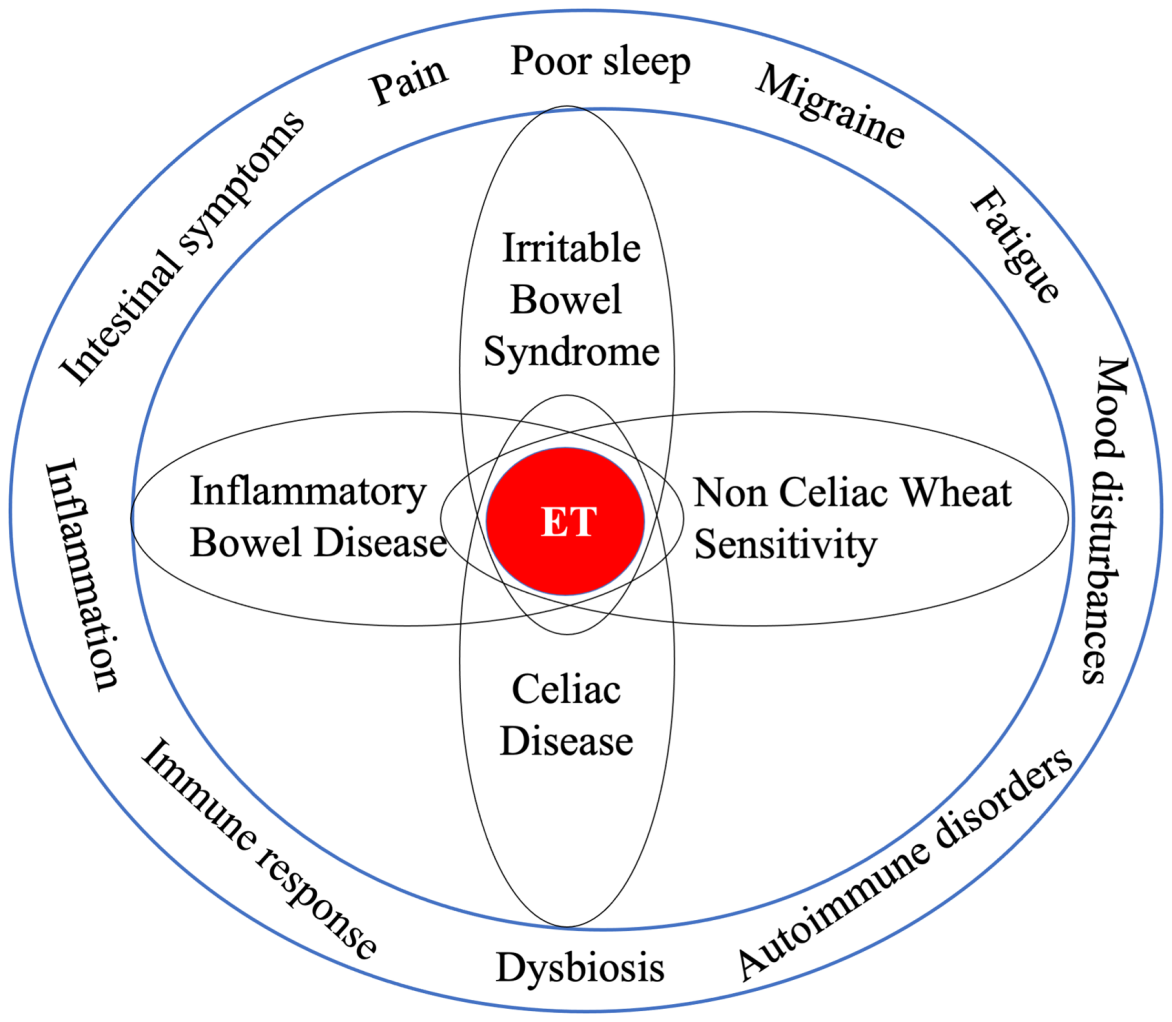
Endometriosis characteristics and symptoms are associated with other disease states, which raises questions about the chance of “shared” underlying pathologies as well as possible risks that one initiates and/or potentiates the other. Since associations are no proof of causality great care should be taken with the interpretation of their meaning.

Available observational studies have suggested a strong overlap between symptoms associated with endometriosis and with other chronic diseases which are also characterized by immune and inflammatory responses such as CD, ulcerative colitis, Crohn’s disease, non-celiac/wheat gluten sensitivity (NCGS/NCWS) and IBS (7, 69–73). In women diagnosed with endometriosis a 2-3fold risk to also be diagnosed with IBS has been observed (38, 39). In women suffering from IBS and being suspect of gluten related symptoms, a 5- to 6-fold risk of suffering from CD was found (74). Both a case control study (75) and a systematic review and meta-analysis (76) concluded that endometriosis was associated with autoimmune diseases including ID and IBD. However, both authors addressed there is little understanding of shared biological mechanisms and pathways between endometriosis and autoimmune diseases that would explain such association (Figure 2).

Because specific food components are involved in the pathologies of CD, ulcerative colitis, Crohn’s disease, NCWS and IBS, and because these diseases are thought to be associated with endometriosis, suggestions have been made that nutritional strategies recommended in these conditions might also be relevant for women diagnosed with endometriosis. A few examples of diets suggested to be beneficial for women diagnosed with endometriosis are an anti-inflammatory diet that is rich in long chain poly-unsaturated fatty acids (PUFA) and polyphenols and low in trans-fatty acids (58, 77–79); the Low FODMAP diet (FODMAP – Fermentable Oligo-, Di-, Monosaccharides And Polyols which give rise to gas formation, osmotic fluid shift, feelings of bloating and intestinal distress) (27), a low nickel diet (80) and finally supplementation with pre-, pro- and synbiotics^2^ (81). In addition, it was advised to reduce or avoid red meat consumption (both processed and non-processed) because of its content of heme-iron (a potential pro-oxidant) and a suggested presence of estrogens due to hormonal treatment of cattle. Hormonal treatment of cattle is a farmers practice to stimulate growth and meat yield, in countries where this is legally allowed like the United States. However, it is forbidden in all European countries (58). The avoidance of red meat is part of the so-called endometriosis diet (37, 82). However, the extensive analysis of available data performed by Shafrir et al. (83) and by Nap and de Roos (30) concluded that “conflicting results have been reported on the associations between endometriosis and dietary components.” Therefore, there is no clear advice regarding dietary interventions in the most recent ESHRE Guideline for endometriosis (1, 30, 83).

Although pain perception may be used as a subjective clinical endpoint it is influenced by many factors, among which also positive and negative expectations, cognitive attention and mood. Nevertheless, patients who self-implement nutritional measures indicate that these result into better wellbeing and less pain perception (28, 33, 37, 40, 82, 84). Part of the positive effect of a food component on mood and QoL may be attributed to a placebo effect (a positive effect experienced just because the user thinks that intake of the component helps the condition) or nocebo effect (belief that a negative outcome occurs because a certain food component causes harm and avoiding it will result in feeling better). Both placebo and nocebo effects are based on expectations which are subject to tremendous influence of the news in social and main stream media as well as opinions of peers and health professionals in close contact. The latter was confirmed in a randomized, double blind, placebo-controlled, international multicenter study, addressing the effects of expectancy on experiencing adverse reactions of gluten intake, after exposure to foods labelled “gluten free” or “containing gluten” (85). It was shown that the combined effect of expectancy had a strong effect on overall and individual GI symptoms, reflecting a nocebo effect. In addition, a recent meta-analysis concluded that evidence of positive impact on pain perception and QoL, was derived from non-randomized controlled trials with small sample sizes. There were multiple sources of bias and when assessed using GRADE criteria the evidence was graded as low to very low-quality (41, 86, 87). Nevertheless, despite the presence of placebo/nocebo effects, Leonardi et al. plead to encourage patients and healthcare providers to more strongly consider and encourage diet treatments if this improves the patients’ wellbeing, even if these improvements may be induced by effects of expectation and belief (84). This strategy may bear certain risks because the advised diet treatment might induce changes in nutrient intake. This is important to patient’s health and is discussed later in this paper.

### Association or proven cause-effect?

#### Endometriosis and microbiota

Although the uterus has been considered a sterile environment for a long time, it has recently been found that the endometrium harbors low quantities of resident microbiota (88). However, much less than in the vagina (89, 90). It has additionally been suggested that fecal compounds and/or microbiota may contaminate the vagina (91) leading to cervico-vaginal dysbiosis, inflammation, infection and tissue damage (92–94). It has been hypothesized that this deteriorates endometrium integrity, allowing for the migration of cells or tissue fragments with blood to other sites. It is suggested that the latter may result in tissue adherence and the initiation of endometriosis lesions (95–98). Similarly, Blander et al. argued that an increase in pro-inflammatory and a decrease in anti-inflammatory microbiota composition may result in conditions leading to increased epithelial permeability, giving rise to microbial infiltration and translocation to blood (99).^3^ This may in turn lead to metabolic inflammatory conditions similar to endometriosis (101, 102).

Dysbiosis in CD, NCWS, ulcerative colitis and Crohn’s disease is associated with inflammation induced impaired intestinal barrier integrity (103) and may lead to passage of microbes into blood, evidenced by the presence of flagellin (marker of microbial translocation from intestine to blood) in blood (104). Because of overlapping inflammatory metabolism and symptoms, it has been speculated that endometriosis and intestinal dysbiosis may interact. However, whether intestinal dysbiosis is causal to developing endometriosis is highly speculative and remains unproven.

#### Intestinal dysbiosis, estrogen cycling and endometriosis

A potential effect of the gut microbiome associated estrobolome—a collection of genes in the intestinal microbiome modulating the amount of resorption of estrogen passing from blood into the intestine - has been described. It was discussed that intestinal dysbiosis may lead to excess estrogens being reabsorbed, causing a hyper-estrogenic environment that will impact endometriosis (64, 105, 106). It has been shown that intestinal resident microbiota communities are influenced by hormonal changes, which behave cyclical. In addition, hormonal fluctuations can modulate anti-microbial peptides in the uterine mucosa and endometrial fluid, as well as influence the local endometriosis immune cells (107, 108). Thus, both the endometrium microbiota composition and the way its metabolism is changed by hormonal cycling and vice versa may theoretically play a role in endometriosis etiology.

Perrotta et al. determined the fecal microbiota composition by collecting rectal swabs of women with and without endometriosis. They found no differences in fecal microbiome composition of endometriosis patients versus their healthy controls (109). However, experimental animal studies suggest that the intestinal microbiota may lead to endometriosis and disease progression. Chadchan et al. observed that antibiotic treatment in an experimental endometriosis mouse model reduced inflammatory markers and endometriosis progression (110). An influencing role of intestinal microbiota related metabolism and/or endotoxemia is a likely explanation. Ni et al. observed that microbiota induced changes in fecal matter of experimental endometriosis mice (increased bile acid and decreased alpha-linolenic acid concentration) were related to disease progression (111). However, in this study dysbiosis and related metabolic effects occurred after the mice were treated with estrogen and endometrial fragments were transplanted. Therefore, the conclusion that dysbiosis was causal to endometriosis development by Ni et al. (111) seems unjustified. How the introduction of endometriosis in these mice has resulted in the initiation of dysbiosis remains unclear. It has been shown that diet and microbiota related increased β-glucuronidase activity can result in elevated estrogens levels whereas a dysbiosis induced decreased activity may induce decreased circulating estrogens (112), which in fact should counteract the earlier suggested positive association between dysbiosis and endometriosis. Neither dysbiosis nor dysbiosis-associated effects on estrobolome, while suggesting that these pose an increasing the risk of endometriosis, should be an argument to avoid gluten. At present, there are no *in vivo* cause-effect data in this respect.

As mentioned above, many associations between the consumption of certain food components and occurrence of disease symptoms are based on observational data showing correlations. However, this does not provide proof of causality. In addition, favorable effects observed after exposure to isolated food components in *in vitro* and in animal intervention studies do not allow direct generalization to daily human mixed diet consumption conditions. Another critical aspect is how well all factors that play a role *in vivo* are truly comparable to the increasingly performed *in vitro* tests. Oral food processing, gastric peristalsis, intestinal transit, the presence of digestion enzymes and organic acids in oral cavity, stomach and intestine (carbohydrases, proteases, gastric acid and bile) as well as the processes of absorption (transport mechanisms) that play a large role in *in vivo* studies, should be similar to the circumstances of *in vitro* studies. However, *in vitro* systems lack feedback from the gastrointestinal sensors in delaying gastric emptying and extending gastric acid exposure. They additionally lack a mucus layer, delaying exposure to the enterocytes for absorption, while at the same time extending intraluminal digestion. In addition, with the exception of organoids, they lack brush border membrane-associated enzyme activities which can influence the rate of digestion and absorption (113, 114).

Another point of concern is that the direct *in vitro* exposure of bioactive molecules to cells or cell-lines are often unrealistic with respect to the *in vivo* situation. The quantitative exposure (bio-accessibility) and the resulting bioavailability may be extremely low *in vivo*. In addition, the test molecules may undergo microbial modification in the intestine prior to absorption. For example, as a result of microbial metabolism, pro-inflammatory and immune stimulating compounds, e.g., conversion of primary to secondary bile acids (115, 116), and alternatively, anti-inflammatory compounds (e.g., derived from antioxidant-polyphenols) (117) may be formed in the intestine. In addition, post-absorption, many of the absorbed compounds will also become subject to conjugation in the liver, which changes their potential bioactivity and health effects. For this reason, total antioxidant capacity, as measured *in vitro*, has no relevance for the *in vivo* situation and its use as “favorable for health” should be discouraged (118).

### Effects of isolated food components vs. mixed diet on endometriosis

Effects of isolated food compounds, as observed in *in vitro* studies, may differ from the effects when it is part of a mixed diet. As an example, it was long assumed that free radicals present in cigarette smoke, cause oxidative stress in the lungs and that this plays a role in the development of lung cancer. Based on *in vitro* data it was thought that supplementing lipophilic antioxidants such as vitamin E and carotenoids would counteract oxidative stress and decrease lung cancer risk. However, a higher lung cancer and death incidence was observed among consumers taking beta-carotene supplements, compared to the placebo group (119, 120).

Positive effects of fruit and vegetable consumption on the risk of developing endometriosis have been suggested because these are rich in antioxidants, micronutrients and dietary fibers (82, 83). However, the observational study by Schwarz et al. (121) drew a contradictory conclusion. After adjusting for a healthy eating index, positive effects associated with fruit fibers disappeared. Even more so, when pooling total vegetable and cruciferous fiber intake an increased endometriosis risk was observed (121). A recent systematic review by Nirgianakis et al. (42) reviewed 9 human and 12 animal studies addressing the effect of dietary interventions on endometriosis. The human studies were classified as supplementation with isolated dietary components (e.g., antioxidants), exclusion or avoidance of selected dietary components (e.g., red meat), or complete diet modification. It was concluded that although the selected animal studies showed promising results, they did not reflect the reality of consumption in humans (42). Accordingly, before making recommendations, effects observed *in vitro* or in animal model studies should always be confirmed by human intervention studies with habitual frequencies and quantities of food intake. Below we aim to put this complexity further in perspective by discussing two specific examples of diet components which, based on experimental data in animals, *in vitro* studies, as well as observational data, are suggested to play a role in endometriosis etiology and/or symptoms, but lack a clear cause-effect evidence: (1) omega-3 fatty acids and (2) red meat. Subsequently, dietary gluten will be discussed in great detail.

#### Omega-3 fatty acids

It has originally been suggested that omega 3 poly-unsaturated fatty acids [*n*-3 PUFA: α-linolenic acid (ALA), eicosapentaenoic acid (EPA) and docosahexaenoic acid (DHA)], as precursors of prostaglandins, express anti-inflammatory activity (122). Observational data show that low fish oil consumption is associated with a 22% more likelihood to be diagnosed with endometriosis (78). Based on this observation supplementation is often advised to patients suffering from inflammatory diseases such as rheumatoid arthritis, IBS, IBD but also endometriosis. However, the question is whether there is a plausible mechanism by which *n*-3 PUFA may either reduce endometriosis initiation or endometriosis associated symptoms.

Supporting that the effects of a single food component may differ from the effect of the component when being part of a mixed diet, studies have shown that the effects of *n*-3 PUFA supplementation may differ for consuming fatty fish as a source of omega 3. Observational study outcomes are conflicting (51, 121, 123), the biological plausibility is lacking and there is debate on the efficacy to reduce inflammation *in vivo* (43, 123–126). Su et al. performed a meta-analysis and concluded that there was no benefit of ALA on reducing blood inflammatory markers (44). In contrast, they surprisingly found that ALA supplementation may increase inflammation. Nodler et al. (127) performed a double-blind placebo-controlled study on the effects of *n*-3 PUFA supplementation for 6 months in adolescent girls and young women with endometriosis. Increased *n*-3 PUFA intake resulted in a reduction in pain scores not different from placebo treatment. It is discussed that “*caution must be applied given the widespread direct marketing of these supplements to girls and women with endometriosis implying a beneficial impact on symptoms. A strong placebo effect was evident in multiple outcome measures, suggesting that participation itself, and not the supplements, conferred improvement even at six months*” (127). Despite the observed association between higher levels of fish oil consumption and reduced endometriosis risk (78), evidence to conclude that *n*-3 PUFA supplementation causally helps to reduce endometriosis initiation or symptoms is lacking. The differing results discussed above reflect that the overall composition of the diet (including other factors influencing inflammatory or anti-inflammatory potential) plays a critical role. In addition, the physical condition of the individual (presence of obesity, associated decreased microbiota diversity and presence of insulin resistance) as well as smoking behavior will also influence the effects of diet and of study outcomes (128).

#### Red meat

A prospective cohort study by Yamamoto et al. (58) investigated the effect of meat consumption on endometriosis risk and found a positive association. They suggested that this may at least partially be caused by heme-iron (acting as pro-oxidant, inflammation inducer) and steroid hormones present in meat. However, other studies did not confirm these findings and suggested that this may only occur when consuming seven or more red meat servings per week, which, with habitual food consumption may only happen in extreme cases (129). These contradicting findings require a critical assessment of potential sources of bias that may influence data interpretation. For example, high meat consumers generally are overweight (130), which is associated with low-grade chronic inflammation (131). Montonen et al. noted that an elevation of inflammation markers (hs-CRP) in high meat consumers was no longer significant after adjusting for overweight (132). In addition, obesity in women, in particular abdominal obesity, is associated with elevated serum and adipose tissue estradiol levels (60, 133–135). It is possible that the effect observed by Yamamoto et al. was due to overweight, which they did not correct for in their analysis (58). Furthermore, Yamamoto et al. referred to other studies suggesting that hormones from steroid hormone-treated animals, present in consumption meat, may play a role in endometriosis risk (58, 136, 137). However, the cited study of Andersson and Skakkebaek (136) concerned pre-pubertal young children whereas in the other study Brinkman et al. (137) studied postmenopausal women. In both cases endogenous estrogen production was low. It is thought that the impact of estrogens from meat consumption on circulating estrogen levels will differ much from that in women of reproductive age, because they have a much higher endogenous estrogen level (137). Concluding, none of the studies available at present quantified estrogen levels in meat (and as a result of consumption) and there is no conclusive data that (red) meat consumption is causally involved in endometriosis etiology and symptomatology.

### Do adverse effects of gluten interact with endometriosis?

Both untreated (newly diagnosed) celiac disease and confirmed endometriosis are associated with inflammation, immune responses and dysbiosis. This raises the question whether other disease states showing similar and overlapping associations may mutually and interactively worsen endometriosis pathology. For example, is it plausible that inflammation and immune response signaling molecules released from intestinal sites due to gluten exposure potentiate inflammation of endometriosis lesions at other sites? To put this matter in a correct perspective, background of gluten, gluten related pathologies and effects of GFD are discussed step by step below.

#### What is gluten?

**Figure 3:**
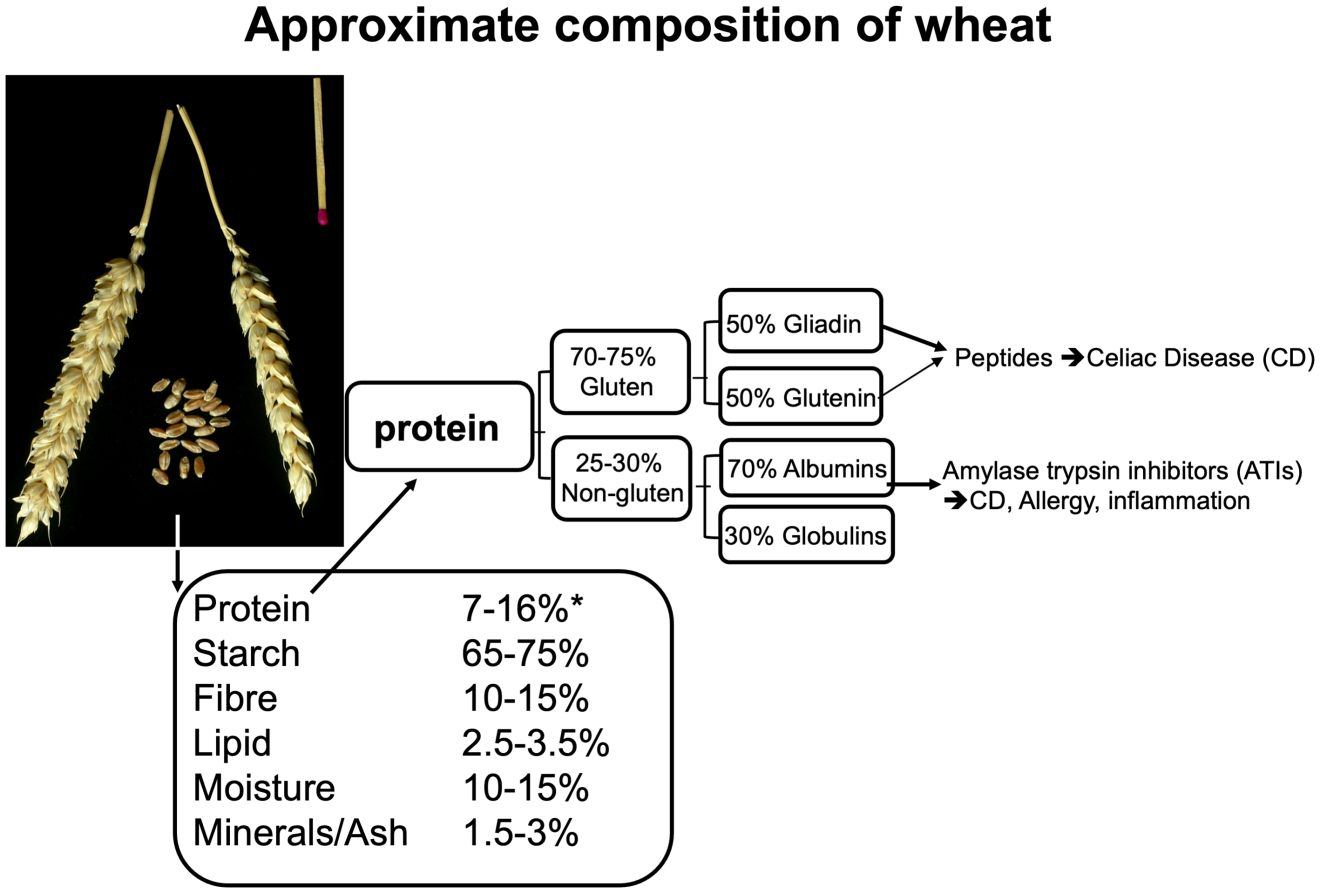
Composition of the wheat kernel and of wheat protein, based on data from (138, 139), adapted from Brouns and Shewry (140).

The main storage proteins of all cereal grains, with the exception of oats and rice, are “prolamins”. This term is based on the fact that these proteins are rich in proline and glutamine. Gluten is comprised of two prolamin fractions, gliadin and glutenin. Figure 3 represents an approximate composition of wheat and wheat protein. Glutenin is particularly important for the formation of an elastic network during dough kneading, subsequently enabling the entrapment of fermentation gases and rising of the dough. Gliadin is the more important fragment, arising from protein digestion (peptides), that can cause adverse reactions in the intestines (see further below). Gluten contributes to about 70–80% of the total grain protein content in common (bread) wheat, ~78% in spelt wheat, ~70% in emmer wheat (all 3 used in bread making) and ~ 77%, in durum wheat (mostly used for pasta making) (141, 142).

Based on the measurement of “true ileal digestibility” it has been suggested that the degree of digestion of wheat protein in humans is ≈90%–95%, similar to the range of digestion of most plant proteins (143). Specific sections of the gluten protein contain proline-rich sequences which cannot be degraded by the human proteases pepsin, trypsin and chymotrypsin. This results in a small fraction of peptides that potentially may cause immune and inflammatory responses in the intestines in susceptible individuals (see next section for detail). The storage proteins in barley (hordein, ~50%–55% of total protein) and in rye (secalin, ~47%–50%) are molecular closely related to gluten and are therefore considered as “gluten proteins” (141, 144). Because avenin proteins in oats do not resemble gluten, oat is generally considered to be a gluten free grain. However, oat-based foods may become contaminated with gluten containing grains or flours in the agri-food chain.

In social media it is often suggested that ancient wheat types contain less gluten and are safer for individuals suffering from gluten disorders. However, an extensive compositional analysis of wheat types showed that over time the protein content slightly declined while starch content increased. Similar to these observations, recent analysis showed that the “ancient” wheat types spelt, emmer and einkorn have higher contents of gluten compared to common wheat (bread wheat) (142). Along with the higher protein content, the amount of gliadin and digestion resistant gliadin derived immuno-reactive peptides involved in the etiology of celiac disease is also higher in these wheats (142, 145–147).

#### Gluten disorders pathology and prevalence

Undigested gluten peptides (size of 10–40 amino acid residues), exposed to the intestinal epithelium, can pass the luminal side of intestinal epithelium and enter the lamina propria *via* the transcellular or paracellular route. Subsequently, peptide deamidation by the enzyme “tissue transglutaminase” (tTG) takes place. In addition, dendritic cells recognize specific amino acid sequences on the surface of the antigen (called epitopes) which can lead to binding to HLA-DQ2 and HLA-DQ8 molecules on antigen-presenting cells and subsequent initiation of immune and inflammatory responses (148). This condition ultimately leads to increased numbers of intraepithelial lymphocytes and villous damage. The latter causes digestion-absorption problems that are found in up to 38% of patients with CD, where one or more nutritional deficiencies (calories, dietary fiber, vitamins and minerals) are seen. This might result in diarrhea, steatorrhea and weight loss (149, 150). Extensive detail on CD etiology and pathology can be found in other reviews (70, 151, 152). Different features of gluten/wheat exposure in CD, wheat allergy (WA) NCWS and of endometriosis are presented in Table 1.

**Table 1:** Features of gluten/wheat exposure in celiac cisease (CD), wheat allergy (WA), non-celiac wheat sensitivity (NCWS), formally often named non-celiac gluten sensitivity (NCGS) and of endometriosis.

	CD	WA	NCWS	Endometriosis
Prevalence	1–2%, Global mean ~1%, more in women than in men (ratio 2:1 to 3:1).	0.25% in global population	0.5–10%? in global population	5–15% in reproductive women, 1.9–5.7% global population
Time interval between gluten/wheat exposure and onset of symptoms	Weeks—years	Minutes—hours	Hours—days	Unclear
Pathogenesis	Autoimmunity (innate and adaptive immunity)	Allergic immune response	Innate immunity	Autoimmunity Unclear (75, 153)
HLA-DQ2/8	HLA-DQ2/8 restricted (~97% positive cases)	Not HLA-DQ2/8 restricted (35–40% positive cases as in the general population)	Not HLA-DQ2/8 restricted (50% DQ2/8-positive cases)	Not HLA-DQ2/8 restricted. No data available in endometriosis patients (154)
Autoantibodies	Almost always present	Always absent	Always absent	Present. HLA class 2 system may be involved in etiology (155)
Enteropathy	Almost always present	Always absent (eosinophils lamina propria)	Always absent (slight increase in IEL)	Often present
Symptoms	Both intestinal and extra-intestinal (not distinguishable between these three gluten-related disorders). Common intestinal symptoms: bloating, abdominal pain, diarrhea, nausea, epigastric pain, alternating bowel habits, dysbiosis, inflammation. Common extra-intestinal symptoms: lack of wellbeing, tiredness, headache, anxiety, foggy mind, impaire quaity of life.			Strong overlap with gluten associated adverse reactions
Complications	Co-morbidities, long-term complications	Absence of co-morbidities, short-term complications (including anaphylaxis)	Absence of co-morbidities and long-term complications (long follow-up studies needed to confirm this)	Co-morbidities, long-term complications

GI, gastrointestinal; GS, gluten sensitivity; IEL, intraepithelial lymphocytes.

Important to notice is that CD only develops in genetically predisposed individuals, expressing the gene haplotypes HLA-DQ2 or DQ8 (156, 157). Approximately 25%–40% of the general population (the exact numbers differ per country that was studied) is predisposed and expresses these gene haplotypes (158, 159). However, based on confirmed diagnosis it is estimated that only about 4%–6% of these predisposed individuals actually develop CD. A global mean population prevalence of about 1% is mostly cited. The fact that the majority of predisposed individuals do not develop CD implies that additional environmental non-gluten factors might play a role in the disease initiation (160).

The prevalence of CD in the global population is about 1%–2%. Santoro et al. observed CD in 5 (2.2%) out of 223 women with confirmed endometriosis whereas only 2 cases of CD were found in the 246 control subjects (0.8%) (161). In addition, Aguiar et al. observed a CD prevalence of 2.5% in a cohort of 120 women with laparoscopically confirmed endometriosis, compared to a prevalence of 0.66% in their control group (162). Based on these observations one may conclude that the actual prevalence of CD among endometriosis patients is only slightly higher than in the general population. Finally, the largest survey thus far was performed in Sweden (163). In this study, using biopsy reports from all Swedish pathology departments, 11,097 women with confirmed CD were identified. None of them had a diagnosis of endometriosis before study entry. These patients were matched with data of 54,992 controls not suffering from CD. All individuals were followed up for a period of at least 5 years. During this period 118 individuals with CD and 399 matched controls developed endometriosis. When restricted to women of reproductive age at study entry (16–45 years), the mean increased risk was 35% (HR 1.35; 95% CI: 1.07–1.69). The authors concluded that endometriosis seems to be associated with previously diagnosed CD. Potential explanations included shared etiological factors and CD-mediated inflammation. How intestinal inflammation would cause initiation of endometriosis remains unclear. However, contrary to previous beliefs, in a recent study by Schwartz et al. (121), gluten intake was associated with a significantly lower risk of endometriosis diagnosis. Although an explanation for this inverse association was unclear, the results provided some evidence that eating gluten-containing foods did not increase risk of endometriosis diagnosis.

A reason often cited for recommending a GFD to endometriosis patients is the study conducted by Marziali et al. (26). They reported that endometriosis symptoms decreased after 12 months of adherence to a GFD. An important aspect of this study is that none of the patients had been tested for CD when being enrolled in the study. When suffering from CD, a GFD will reduce inflammation and immune response related symptoms, which are known to overlap with endometriosis-related symptoms. It may be that individuals showing improvement of symptoms were, at least partly, undiagnosed CD patients. In addition, 2 weeks after starting a GFD, 88 study participants had withdrawn because of side effects caused by a GFD, such as abdominal symptoms due to other food intolerances (which were not specified). Only the 207 patients who showed improvement of symptoms were allowed to continue the study. This approach may have caused undesired skewing into the direction of the favourable effects of a GFD on endometriosis-related symptoms which were seen in 156 participants whereas 51 participants showed no effect. In addition, based on similar effects seen in patients suffering from gluten/wheat sensitivity, as discussed above, strong nocebo effects related to gluten intake and placebo effects due to avoidance of gluten may have been present. Recently it was shown that, compared to non-patients, women suffering from endometriosis were more likely to self-implement a GFD, similar to observations in patients with IBS. In addition, it was six times more likely that women who implemented a GFD also made other dietary changes in the past years (164). An overview observed associations between gluten, CD and endometriosis is given in Table 2.

**Table 2:** Observed associations between gluten, celiac disease (CD) and endometriosis.

Author	Year	Ref	Main finding	Mechanism
Aguiar et al.	2009	(162)	CD in 2.5% in of women with laparoscopy confirmed endometriosis, vs. 0.66% in control group	Unclear
Stephansson et al.	2011	(163)	In women of reproductive age having CD the mean increased risk of developing endometriosis was 35%	Unclear
Marziali et al.	2012	(26)	Endometriosis symptoms decreased significantly after 12 months gluten free diet	Unclear
Caserta et al.	2014	(165)	Case report emphasizing the possible association between CD, endometriosis and infertility	Unclear
Santoro et al.	2014	(161)	CD in 2.2% out of women with confirmed endometriosis vs. 0.8% in control group	Unclear
Schwartz et al.	2022	(121)	Gluten intake is associated with a significantly lower risk of endometriosis diagnosis	Unclear

#### Is dysbiosis in celiac disease a cause or a consequence of the disease?

Zafeiropoulou et al. showed that the presence of specific bacteria was CD dependent despite the fact that adherence to a GFD had altered intestinal microbiota composition (166). Recently it was shown that the abundance of several species, pathways and metabolites was altered at the time of CD diagnosis, characterized by complex patterns of increased pro-inflammatory species and decreased protective/anti-inflammatory species (167). El Mouzan et al. studied 40 children with newly diagnosed CD, 20 healthy controls, and 19 non-CD controls. They observed a significant difference in fecal microbial composition of the CD children, among which increased *Bacteroides* and decreased *Lactobacillus* genus counts (168). Palmieri et al. collected fecal specimens from 46 CD individuals following a GFD for at least 2 years and 30 specimens from healthy controls. The microbial composition of the CD subjects was decreased in *Bifidobacterium longum* genus and several species belonging to the *Lachnospiraceae* family. Also, *Bacteroides* genus was found to be more abundant. In contrast to other suggestions, microbial profiles of the CD patients were consistently “non-dysbiotic” (169). After a systematic review of cross-sectional studies by Kaliciak et al. it was concluded that due to a lack of taxonomic uniformity between studies and the enormous inter-individual differences in microbiota composition, a comparison of microbial communities between CD patients, CD-GFD patients and untreated CD patients was impossible (170). Vacca et al. came to a similar conclusion (171). These recent observations suggest that unfavorable intestinal microbiota changes and associated metabolism can result from various environmental conditions preceding the diagnosis of CD. Examples of these are self-implemented measures to help alleviate experienced gastrointestinal discomfort and pain (e.g., FODMAP avoidance resulting in low fiber intake, GFD, use of nonsteroidal anti-inflammatory drugs and of antibiotics). Although by adhering to a GFD a partial normalization may occur, the effects of GFD on microbiota changes will not only depend on the avoidance of gluten but also significantly on the compositional quality of the GFD (see further below for more detail).

#### Non celiac gluten sensitivity

When hearing or reading from influencers and celebrities on social media that gluten in general can cause health-related harm, one may impose a self-belief that gluten is the cause of a range of intestinal symptoms and general malaise and that these symptoms will disappear after adhering to a GFD. This, as well as associated gluten-free marketing claims, have led to an increasing belief that ‘living gluten-free’ is equal to a healthy lifestyle (172). Within the general population the prevalence of individuals believing that gluten causes harm, resulting in them avoiding gluten, may range from a few percent up to 15% of the general population (172–174). However, the actual number of individuals having symptoms after consuming gluten or wheat containing foods, while not suffering from CD, wheat allergy or dermatitis herpetiformis, appear to be much lower.

For example, Capannolo et al. studied 392 persons (307 females and 85 males) selected from an IBS cohort. They had contacted the gastroenterology unit because they experienced gastrointestinal symptoms, particularly after consuming gluten. During their initial CD screening, it was found that the positive predictive value of the gluten-related symptoms (defined as the probability that someone with symptoms actually suffers due to gluten/wheat exposure) was only 7% (74). To put the latter in a correct prevalence perspective, 7% of an IBS cohort (usually representing 10%–12% of total population) would equal a NCWS prevalence of close to 1% in the total population. Molina-Infante and Caroccio analyzed data from 10 double-blind, placebo-controlled, gluten-challenge trials, covering data from 1.312 adults. They observed gluten specific effects in 38 of 231 self-reported NCWS patients, confirming the low prevalence of proven gluten/wheat disorders in people reporting symptoms after gluten/wheat consumption. In addition, a 40% rate of nocebo effects was observed (175). However, it should be noted that the percentage of individuals who were newly diagnosed to suffer from CD during the initial study patient entry screening was 6.63% in the study of Capannolo et al. (74). This percentage is much higher than the general population prevalence of about 1% and warrants diagnosis to confirm or reject the presence of CD in both IBS and endometriosis patients.

#### Effects of non-gluten compounds on symptoms overlapping with endometriosis

In studies applying challenge tests with gluten or FODMAP it was observed that individuals with self-reported gluten sensitivity reacted primarily to the rapidly fermentable fructans present in grains. Not gluten but fermentation of FODMAP was found to be a significant trigger of IBS and NCWS symptoms that showed an overlap with endometriosis symptoms (176–178). Crawley et al. (178) performed a double-blind placebo-controlled gluten-food challenge with equal cross-over, in a population cohort of 1,266 participants (aged 15–21 years). They studied participants who reported gluten induced gastrointestinal symptoms, as indicated by self-reported questionnaires. They found that, compared to placebo, adding gluten to the diet neither induced gastrointestinal symptoms nor worsened mental health. A high nocebo effect was noted. In fact, their gastrointestinal symptoms reported were similar to IBS symptoms which may be caused by multiple factors (178). These findings do not exclude the possibility that a small subset of individuals reporting sensitivity to gluten/wheat may suffer from an intestinal non-classical type of food allergy (not associated with immunoglobulin E). In this condition, systemic immune activation and compromised intestinal epithelial barrier integrity have been found (104, 179). It is thought that amylase trypsine inhibitors (ATIs), present in wheat and in commercially used wheat gluten isolate, may play a crucial role here (180–182).

### Specific dietary measures and their potential consequences

#### Gluten free diet

Following a life-long GFD is challenging, costly and can be socially isolating. Children and adults with CD report reduced QoL because of the lifelong dietary restrictions (183). The availability of gluten free food (all food without wheat, barley, rye and for food production technological reasons added gluten isolate) is still very limited. In addition, commercial gluten free food is often of doubtful composition. The use of flours from corn, rice, potato and tapioca, in exchange for gluten containing sources, results in a reduced content of micronutrients, dietary fiber and protein and a higher glycemic response. In addition, there is a higher use of saturated fat, trans-fat and salt in the processing of gluten free foods (150, 184, 185) and often gluten free labeled foods do still contain significant amounts of gluten (186, 187).

A low dietary fiber intake is of particular concern (150) because it will minimize fiber fermenting microbiota diversity (in particular those from *Bifidobacterium* and *Lactobacillus* genera (188)) and metabolism which play a key role in immunity and health (169). Based on a population-based study with 124,447 participants, Littlejohns et al. concluded that GFD followers have a poorer self-reported health and a higher prevalence of blood and immune disorders and undesired digestive conditions (164). Because adherence to a GFD and/or FODMAP diet has potential risks of low fiber intakes, special attention to educate and guide endometriosis patients to help select appropriate fiber rich foods is needed. For extensive reviews of these aspects see (189–191).

#### Low FODMAP diet

For patients suffering from IBS and other diseases associated with IBS-like symptoms it is often recommended to reduce the consumption of FODMAP. Within this category of rapidly fermentable carbohydrates, fructans present in grain-based foods such as bread may lead to a significant daily FODMAP intake (192). However, the recommendation to avoid these may result in an important reduction of overall fiber intake, unless compensated by consumption of other high fiber sources (193). It has been shown that chronic low intake of soluble, well-fermentable fiber (diet low in plant-based foods) is associated with small intestinal and colonic microbiome disturbances, decreased intestinal mucus layer thickness and impaired barrier function/increased permeability. This is based both on a study where a gnotobiotic mouse model where mice were colonized with synthetic human intestinal microbiota was applied and an extensive systematic review including only human studies (194, 195). More importantly, low fiber intake causes a reduced saccharolytic fermentation in the colon, resulting in low production of short chain fatty acids (SCFA) - most importantly butyrate, propionate and acetate. These SCFA are essential for immunity and health protection of the intestine. A reduced SCFA production is known as a pro-inflammatory condition and is associated with an increased risk of IBD, intestinal cancer, reduced insulin sensitivity, intestinal tract associated immune compatibility, impaired intestinal barrier function and/or enhanced permeability. In addition, during reduction of saccharolytic fermentation, an increased protein fermentation and formation of hydrogen sulfide, ammonia, and *p*-cresol and branched-chain fatty acids takes place. These changes are known to promote intestinal inflammation and are also implicated in the etiology of intestinal cancers (196, 197). Hill et al. concluded that a low FODMAP strategy should be implemented with care due to the psychological and nutritional risks of a restrictive diet (198). Before recommending a complex and restrictive diet, such as the low FODMAP diet or a GFD, a positive medical diagnosis is a must (165, 199). When in post diagnostic guidance the patient is properly educated and guided long-term by nutrition experts, these risks can be minimized (200).

### Limitations of available studies

Currently available systematic reviews and meta-analyses were all done in the same period (2020–2021), thus considered to have the same available database. In general study outcomes presented and discussed in these reviews showed positive, negative or no associations between the consumption of certain foods and/or nutrients and prevalence of endometriosis, or the perception of pain and QoL. Most intervention studies were of relatively short duration, did not have appropriate control conditions, were subject to significant bias and resulted in low to very low scores of evidence quality. The latter was also concluded in systematic reviews of observational data. As a result, interpretations about the effects of specific foods and/or food components on endometriosis were equivocal (128, 129, 132, 144). More recently, Nap and the Roos concluded in their critical review that evidence regarding the efficacy of dietary interventions in women with endometriosis is conflicting and effects of dietary interventions on specific types of endometriosis (e.g., superficial peritoneal endometriosis, ovarian endometrioma and deep infiltrating endometriosis) remains unknown. In addition, it is conceivable that certain co-morbidities (e.g., obesity) may also influence the effect of dietary interventions. Moreover, they stated that evidence regarding plausible biochemical mechanisms, resulting in the perceived effects of the dietary intervention, is scarce or lacking (30). As result, it is impossible to make evidence-based recommendations for dietary measures at present, to help reduce the initiation of endometriosis and its management once diagnosed. Current healthy eating guidelines, which include avoiding trans fatty acids, limiting alcohol, meat and salt consumption should also be followed by endometriosis patients (see further below). According to the conclusions presented in Table 3 there seems to be consensus that there is a clear need for better quality studies.

**Table 3:** Conclusions and recommendations related to moderate-high risks of bias and low to very low-quality evidence in observational and intervention studies evaluated in systematic reviews/meta-analysis.

Author	Year	Ref	Conclusions and recommendations
Helbig et al.	2021	(86)	Analyzed 19 studies. Results currently available do not permit a clear, scientific recommendation or indicate a detailed diet. To be able to derive more concrete recommendations, we require further studies to investigate the influence of nutrition on endometriosis.
Huijs and Nap	2020	(40)	Study (*n* = 12) quality, including risk of bias, was assessed using GRADE criteria and all were of low to very low quality. Future studies are necessary to gain evidence about which food products are effective and in which amounts.
Nirgianakis et al.	2022	(42)	All animal (*n* = 12) and human (*n* = 9) studies analyzed were characterized by moderate and/or high-risk bias limiting the validity of the results. More and higher quality original studies are urgently needed to enable safe conclusions on this topic.
Osmanlioglu and Sanlier	2021	(87)	Analyzed 25 human studies and 7 animal studies. Due to the limited size of the samples significance of the association between diet and endometriosis is not conclusive. (Grading of evidence quality was not presented). Further research is needed to better identify the role of diet on endometriosis.

### Qualitative dietary guidelines for endometriosis patients

A regular consumption of whole grain foods is strongly associated with significant reductions in the risks of diabetes, cardiovascular disease, intestinal inflammatory disorders, intestinal dysbiosis, colon cancer as well as a more favorable weight management. The quantity of dietary fibers and associated bioactive molecules play an important role in this (102, 201–212). These observations have resulted in a globally supported recommendation of food authorities to regularly consume whole grains as part of a healthy diet and lifestyle. Accordingly, there are good reasons to advise the consumption of grains (most of which do contain gluten and FODMAP) to endometriosis patients when there is no diagnosis of biopsy-proven gluten disorders and/or IBS-related FODMAP fermentation distress.

In general, women with endometriosis should be recommended to follow the qualitative dietary guidelines as adviced by the World Health Organisation (WHO), encompassing restriction of salt, red and processed meat products, reduction of added sugars, syrups and refined carbohydrate/starch sources. In addition, regular intake of whole grains, fruits/vegetables, choice of fat sources rich in mono/polyunsaturted fatty acids and a reduction of foods containg trans fatty acids (209, 213–216). These recommendations are associated with reduced diet related inflammation and significant reductions in chronic disease. Education of endometriosis patients in this respect will help to avoid implementation of self-treatment strategies, some of which in long term may result in negative health outcomes, particulalry when not professionally guided.

## Conclusion

Endometriosis is a multifactorial disorder of still largely unknown etiology. Many etiological and causality hypotheses have been put forward. Yet, none of these give a satisfying full evidence-based explanation for the various manifestations of the disease. A role of nutrition related metabolic, inflammatory and immune factors has been proposed to play a role in the etiology and the symptomatology but this is largely based on assumptions rather than good quality evidence. Endometriosis can be associated with multiple symptoms that strongly overlap with those of CD, Crohn’s Disease, NCWS and IBS. They typically share common pathological factors such as inflammation, immune responses, microbiota dysbiosis and impaired epithelial barrier function. In addition, chronic symptoms of poor sleep, chronic fatigue, changed mood, anxiety, migraine and depression are encountered. This, in addition to high recurrence rates, often lead to patient treatment unsatisfaction and the search for alternative, non-medical strategies to help relief symptoms. Among these are diet related recommendations with the target to help reduce inflammation and pain.

Patients reported that by applying dietary interventions, they felt better and their QoL improved. However, a careful and critical analysis of the scientific evidence regarding the observed positive effect of dietary interventions on symptoms and QoL points to a strong influence of placebo and nocebo effects. Observational data linking diet factors to the risk of endometriosis are conflicting and form no evidence of causality. Effects of nutrients and/or food components largely depend on molecular characteristics, which may differ significantly within a certain class. The generalizing that a certain class, e.g., “dietary antioxidants”, helps reduce oxidative stress and inflammation, and is therefore beneficial to reduce endometriosis symptoms, remains unproven. Claims that the inclusion or exclusion of certain nutritional components is beneficial for endometriosis prevention and management are largely based on data from experimental *in vitro* or animal studies that suggest positive effects. However, these do generally not reflect habitual diet practices and could be misleading.

There is currently no data available of longer-term nutritional intervention studies using established biomarkers of well-defined clinical endpoints of pathology and symptoms. The fact that grains containing gluten, ATIs and FODMAP are involved in the etiology of CD, NCWS and WA, of which symptomatology and a number of pathological factors overlap with endometriosis, does not provide evidence of a causal role in endometriosis etiology. In addition, there is no convincing evidence that the prevalence of CD, NCWS or IBS is increased in endometriosis patients. Given the potential impact that adhering to a life-long GFD may have on QoL, nutritional status, intestinal microbiota composition and potentially health, a GFD recommendation to endometriosis patients should be discouraged, unless a patient has been tested and positively diagnosed to suffer also from a gluten related disorder.

## Author contributions

FB: wrote the initial draft of the manuscript and conceptualized and prepared figures. AH, VM, JM, DK, and KV: wrote parts belonging to their expertise. FB, AH, DK, KV, MB, JM, and VM: contributed to revising and editing the manuscript. All authors have revised the final version of the manuscript and approved of the submitted manuscript.

## Funding

Open access costs for this publication were funded by Maastricht University and Amsterdam UMC. Maastricht university receives funding from the Dutch Government “TKI- Top Knowledge Institute,” ref. nr TKI1601P01 joint funded by Agri-Food chain businesses who donated unrestricted research grants for the international research consortium “Well On Wheat?,” addressing the study of grain based foods and gluten related disorders. Donating industries have no influence on study execution, interpretation/publishing of data and conclusions drawn (https://www.wellonwheat.org).

## Conflict of interest

The authors report to have received funding for attending/speaking at conferences and/or other funding that might be perceived as potential conflict of interest, as follows: FB: travel and speaker fees in the period 2018–2020 for various conferences addressing health aspects of cereals and bread with special reference to gluten disorders in Brussels, Paris, Cappadocia, Athens, Munich, Istanbul, Wageningen, Leuven and Barcelona. AH: travel grant from Merck KGaA to visit the congress of the European Society of Human Reproduction and Embryology (ESHRE) 2022 in Milano. DK: research funding from Allergan, Will Pharma, Grunenthal, ZonMw, MDLS, UEG, Horizon 2020, Rome Foundation, all outside of submitted work. His institution has received speaker’s fee from Dr. Falk on his behalf, outside of submitted work. JM: research funding from Maxima Medical Center and ZonMw, both outside of submitted work. VM: travel and speaker’s fees from Guerbet as well as research grants from Guerbet, Merck and Ferring, all outside of submitted work.

The remaining authors declare that the research was conducted in the absence of any commercial or financial relationships that could be construed as a potential conflict of interest.

## Publisher’s note

All claims expressed in this article are solely those of the authors and do not necessarily represent those of their affiliated organizations, or those of the publisher, the editors and the reviewers. Any product that may be evaluated in this article, or claim that may be made by its manufacturer, is not guaranteed or endorsed by the publisher.

## Footnotes

^1^Dysbiosis is a lasting unfavorable change in the composition/diversity of resident microbiota communities and their metabolism, relative to that found in healthy individuals (60).

^2^Prebiotics: fermentable carbohydrates that are preferably fermented by health beneficial microbiota such as lactobacilli and bifidobacteria. Probiotics: food supplements supplying health beneficial live microbiota. Synbiotics: supplement containing pre- and probiotics.

^3^Microbial translocation is defined as the passage of both viable and nonviable microbes and microbial products such as endotoxin, across an anatomically intact intestinal barrier (100).

## Appendix

For this narrative overview, research and review papers were obtained using the university E-Library academic search tools PUBMED, WEB OF SCIENCE, SCOPUS, EMBASE and ENDNOTE global search tool. Search terms used were: endometriosis, diet, dietary intervention, nutrient supplementation, self-management, adjunct therapy, inflammation, immune response, microbiota, dysbiosis, irritable bowel syndrome, inflammatory bowel disease, celiac disease, intestinal permeability, leaky gut, gluten, FODMAP, gluten sensitivity, non-celiac wheat sensitivity, QoL migraine, mood, anxiety, sleep, fatigue, nocebo effects, placebo effects, polyunsaturated fatty acids, PUFA, omega 3, short chain fatty acids, SCFA, meat, fish, food estrogens, whole grain, dietary fiber, antioxidants, polyphenols, gluten free diet. In research papers and scientific reviews the reference lists were checked for additional sources and relevant interpretations and related citations were cross-checked with the content/data in the original sources cited.
