# Corrigendum: Diet associations in endometriosis: a critical narrative assessment with special reference to gluten

**DOI:** 10.3389/fnut.2023.1295983

**Published:** 2023-11-06

**Authors:** Fred Brouns, Annelotte Van Haaps, Daniel Keszthelyi, Koen Venema, Marlies Bongers, Jacques Maas, Velja Mijatovic

**Affiliations:** ^1^Department of Human Biology, School for Nutrition and Translational Research in Metabolism, Maastricht University, Maastricht, Netherlands; ^2^Endometriosis Center, Amsterdam University Medical Centers, Academic Medical Center, Amsterdam, Netherlands; ^3^Amsterdam Reproduction and Development Research Institute, Amsterdam, Netherlands; ^4^Division of Gastroenterology-Hepatology, Department of Internal Medicine, School for Nutrition and Translational Research in Metabolism, Maastricht University, Maastricht, Netherlands; ^5^Centre for Healthy Eating & Food Innovation (HEFI), Maastricht University, Maastricht, Netherlands; ^6^Department of Obstetrics and Gynecology, Máxima Medical Center, Veldhoven, Netherlands; ^7^Grow-School of Oncology and Reproduction, Maastricht University, Maastricht, Netherlands; ^8^Department of Obstetrics and Gynaecology MUMC+, Maastricht, Netherlands

**Keywords:** endometriosis, diet associations, diet recommendations, omega-3, red meat, gluten-free

In the published article, there was an error. A correction has been made to the Abstract

This sentence previously stated:

“This includes the avoidance of red meat and omega-3, an increasing intake of foods rich in anti-oxidants, micronutrients and dietary fibers (e.g., fruit, vegetables) and the appliance of a gluten free diet.”

The corrected sentence appears below:

“This includes the avoidance of red meat, an increasing intake of foods rich in anti-oxidants, omega-3, micronutrients and dietary fibers (e.g., fruit, vegetables) and the appliance of a gluten free diet.”

The authors apologize for this error and state that this does not change the scientific conclusions of the article in any way. The original article has been updated.

